# Correction: Complex networks applied to political analysis: Group voting behavior in the Brazilian congress

**DOI:** 10.1371/journal.pone.0330648

**Published:** 2025-08-20

**Authors:** Tiago José de Oliveira Toledo Junior, Diego Raphael Amancio, Roseli Aparecida Francelin Romero

In the Funding section, the grant number from the funder São Paulo Research Foundation is missing. The grant number is: 2020/06271-0.
