# Supplementary figures and images for: Complex networks applied to political analysis: Group voting behavior in the Brazilian congress
Source: PLoS One. 2025 Apr 14;20(4):e0319643. doi: 10.1371/journal.pone.0319643 (PMC11996218; doi:10.1371/journal.pone.0319643)

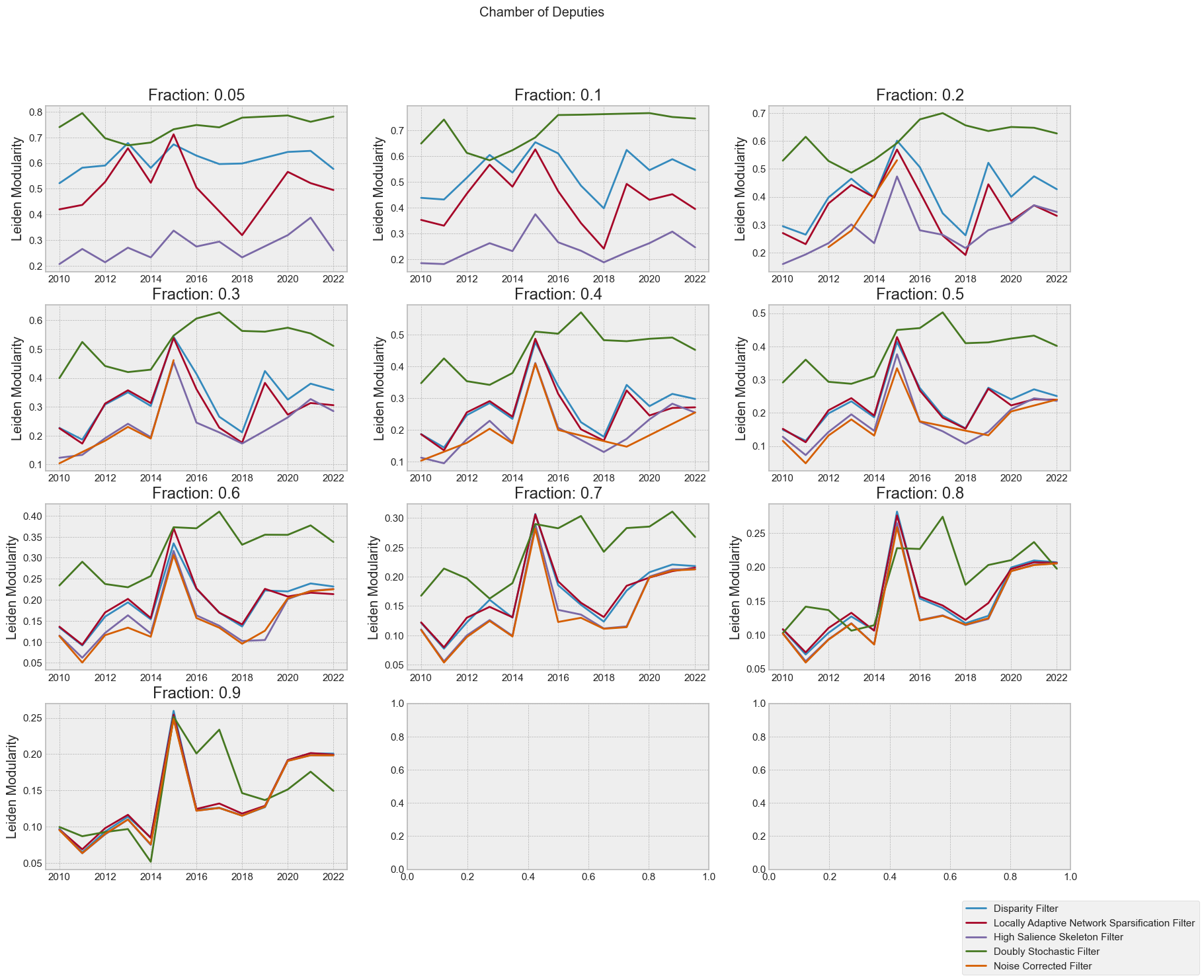

Supplement: S1 Fig — (TIF) [file pone.0319643.s002.tif]

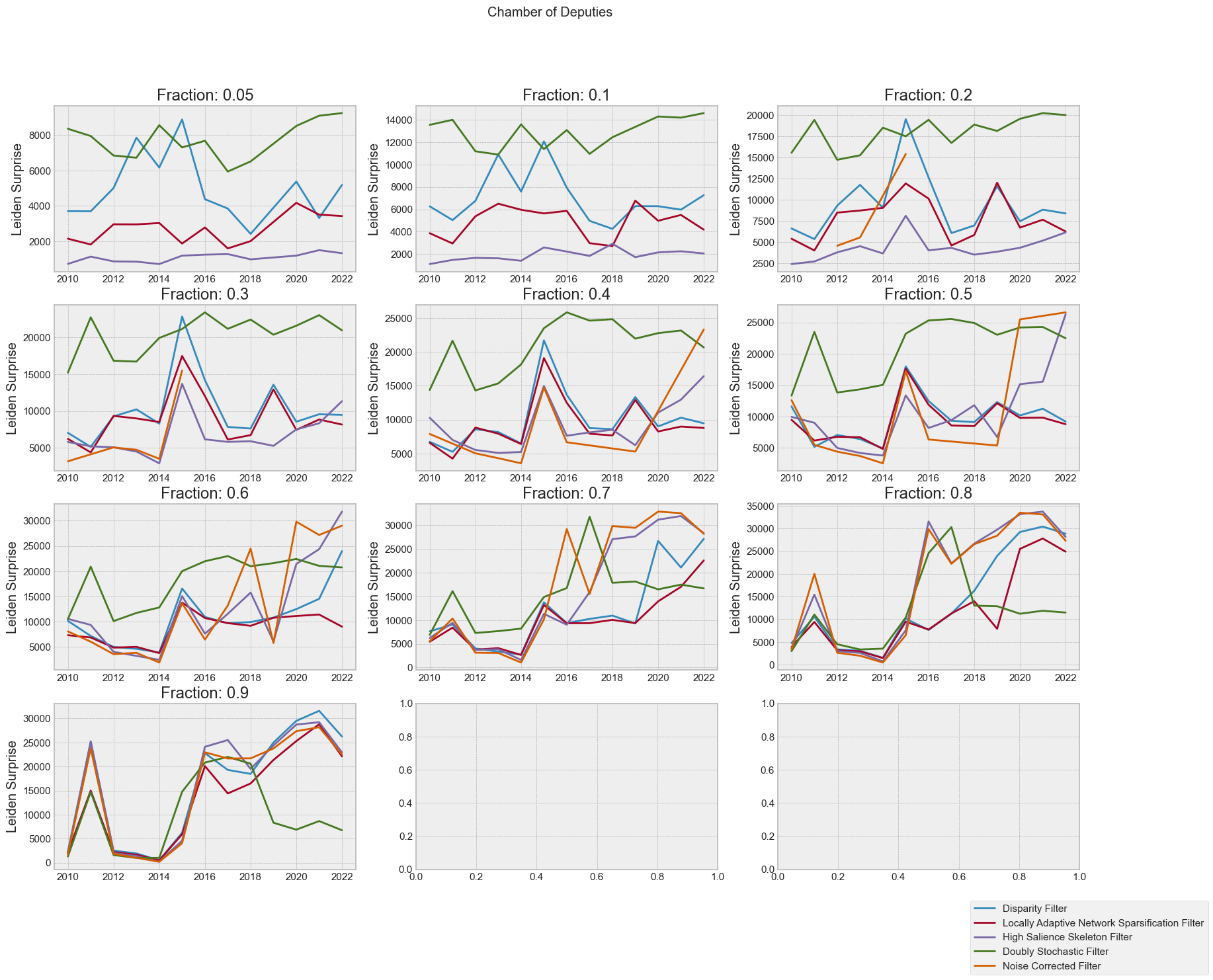

Supplement: S2 Fig — (TIF) [file pone.0319643.s003.tif]

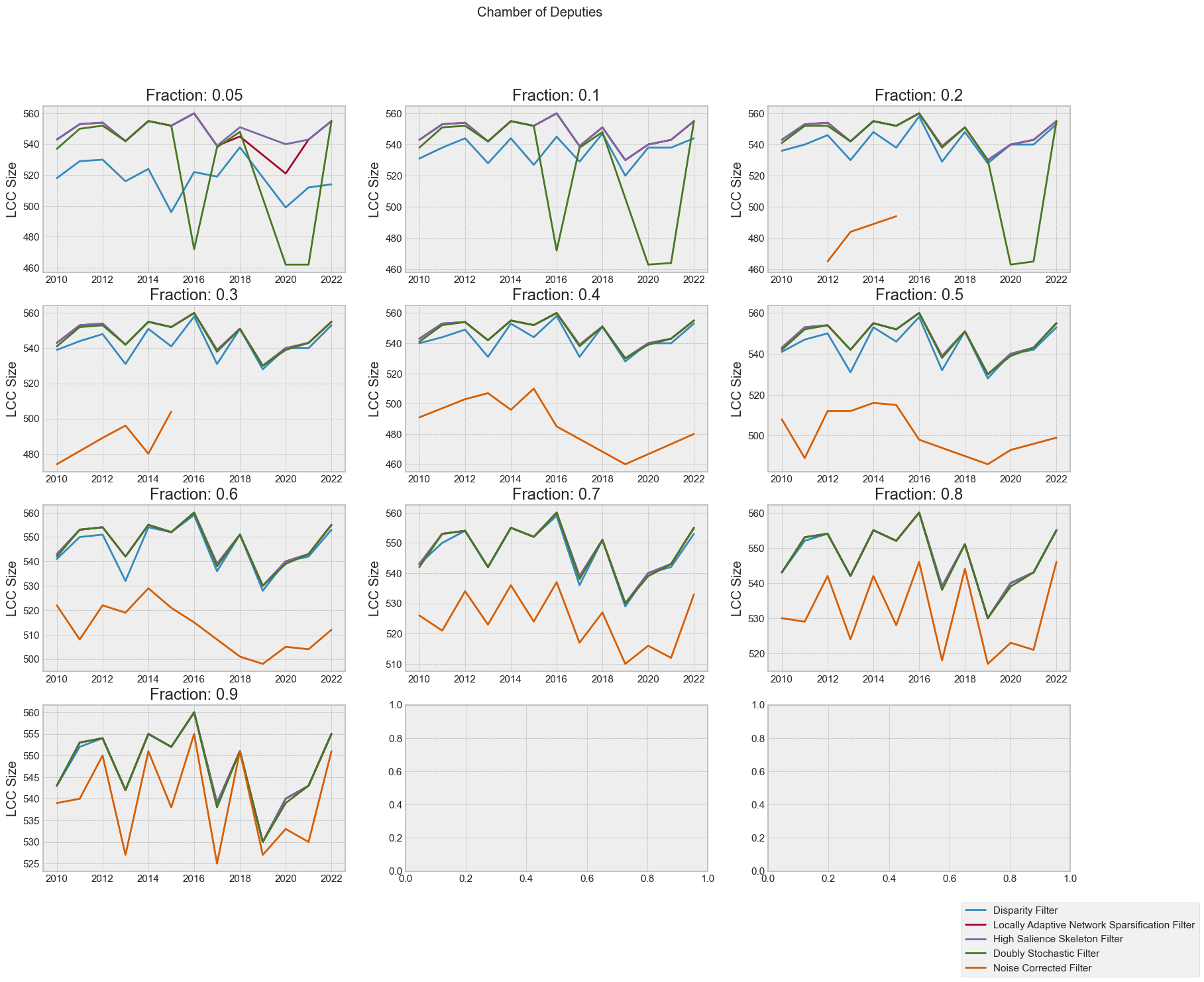

Supplement: S3 Fig — (TIF) [file pone.0319643.s004.tif]

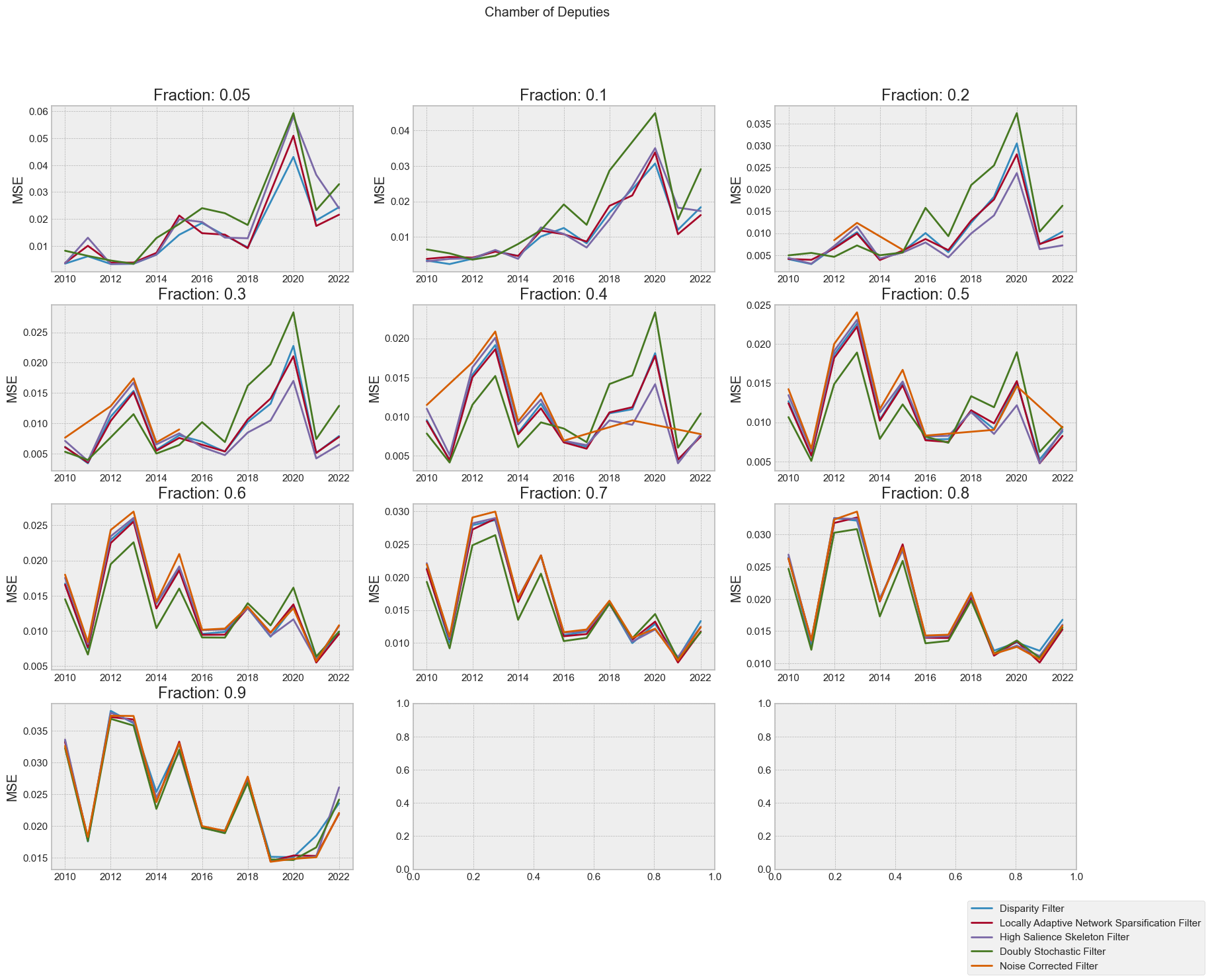

Supplement: S4 Fig — (TIF) [file pone.0319643.s005.tif]

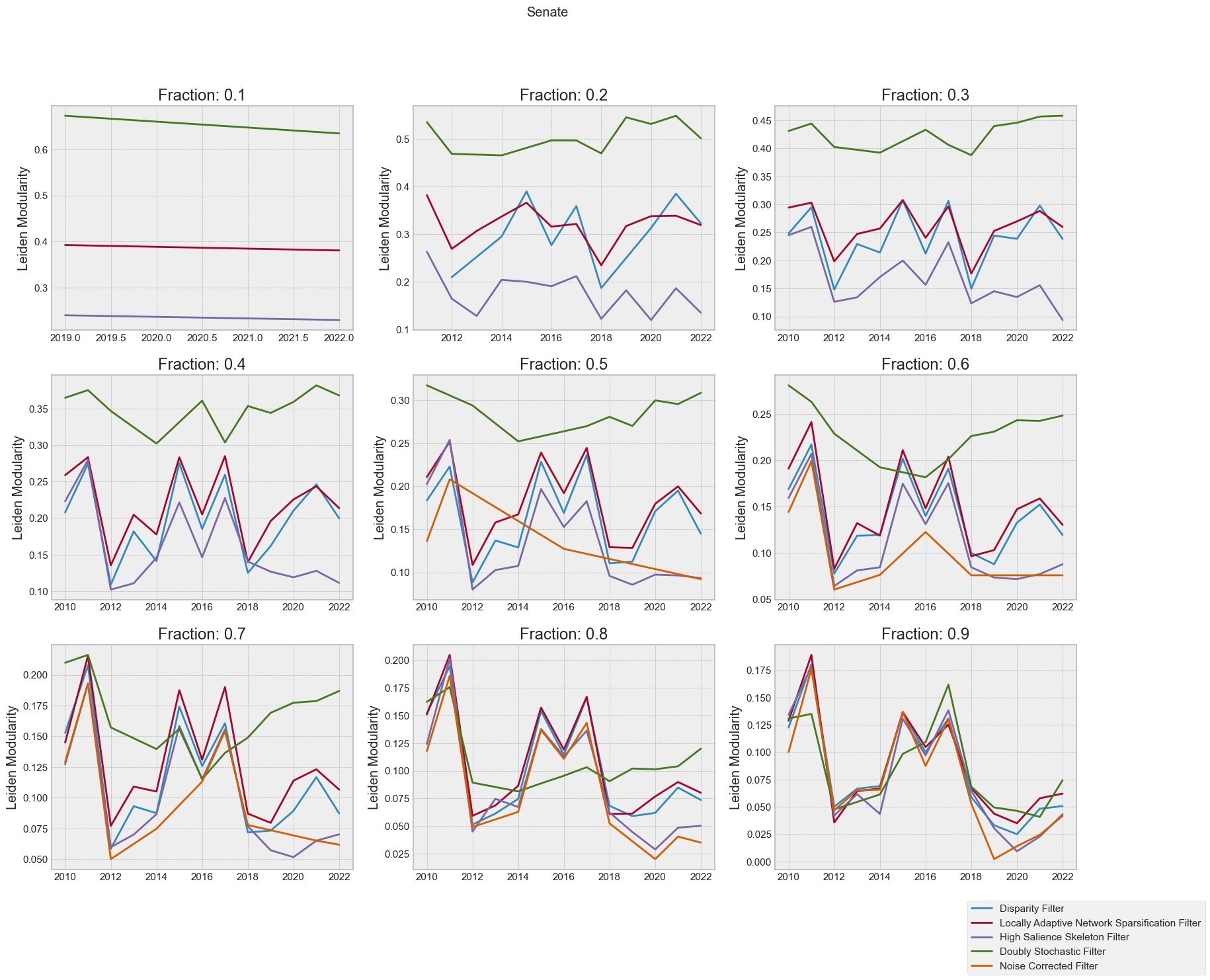

Supplement: S5 Fig — (TIF) [file pone.0319643.s006.tif]

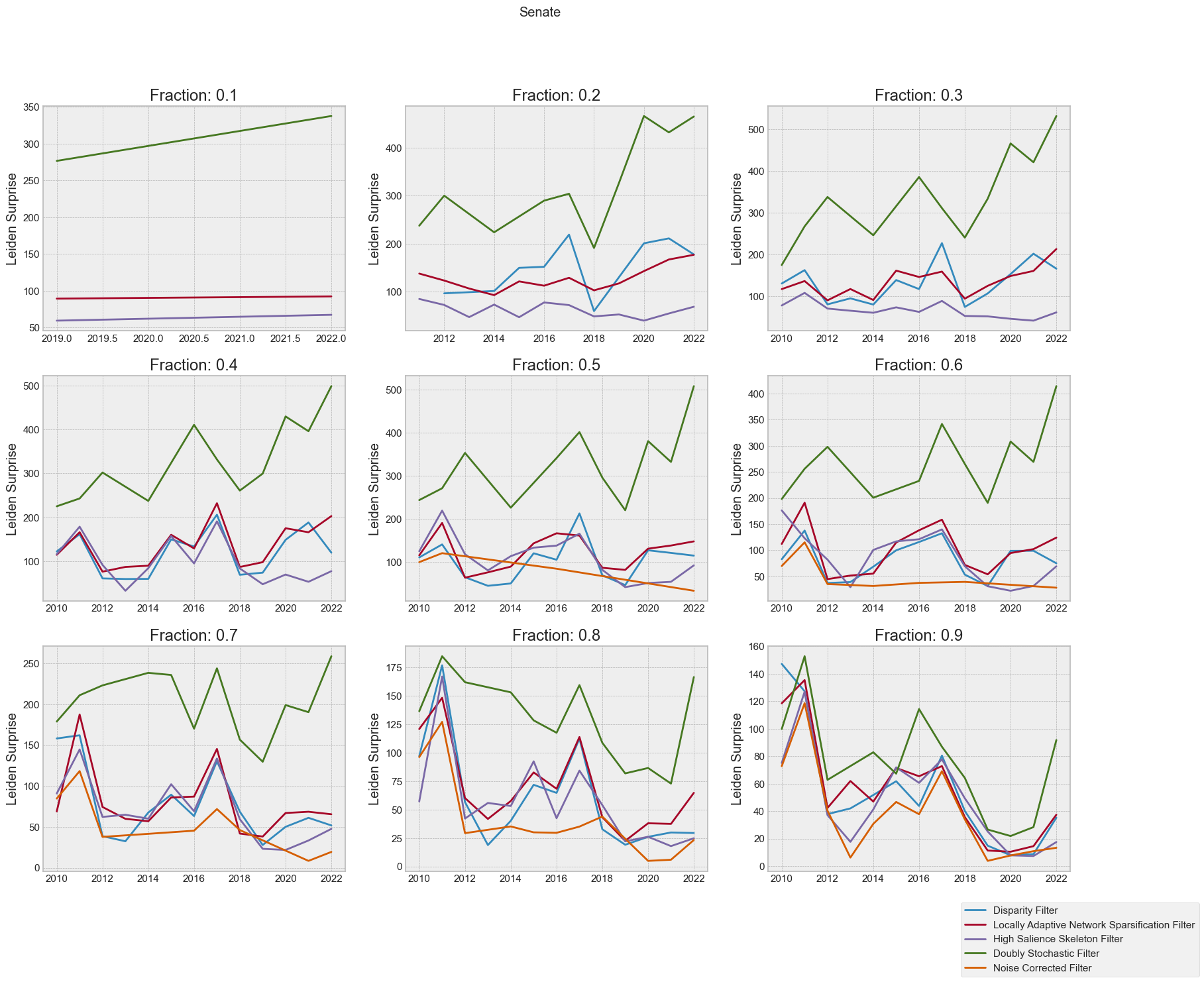

Supplement: S6 Fig — (TIF) [file pone.0319643.s007.tif]

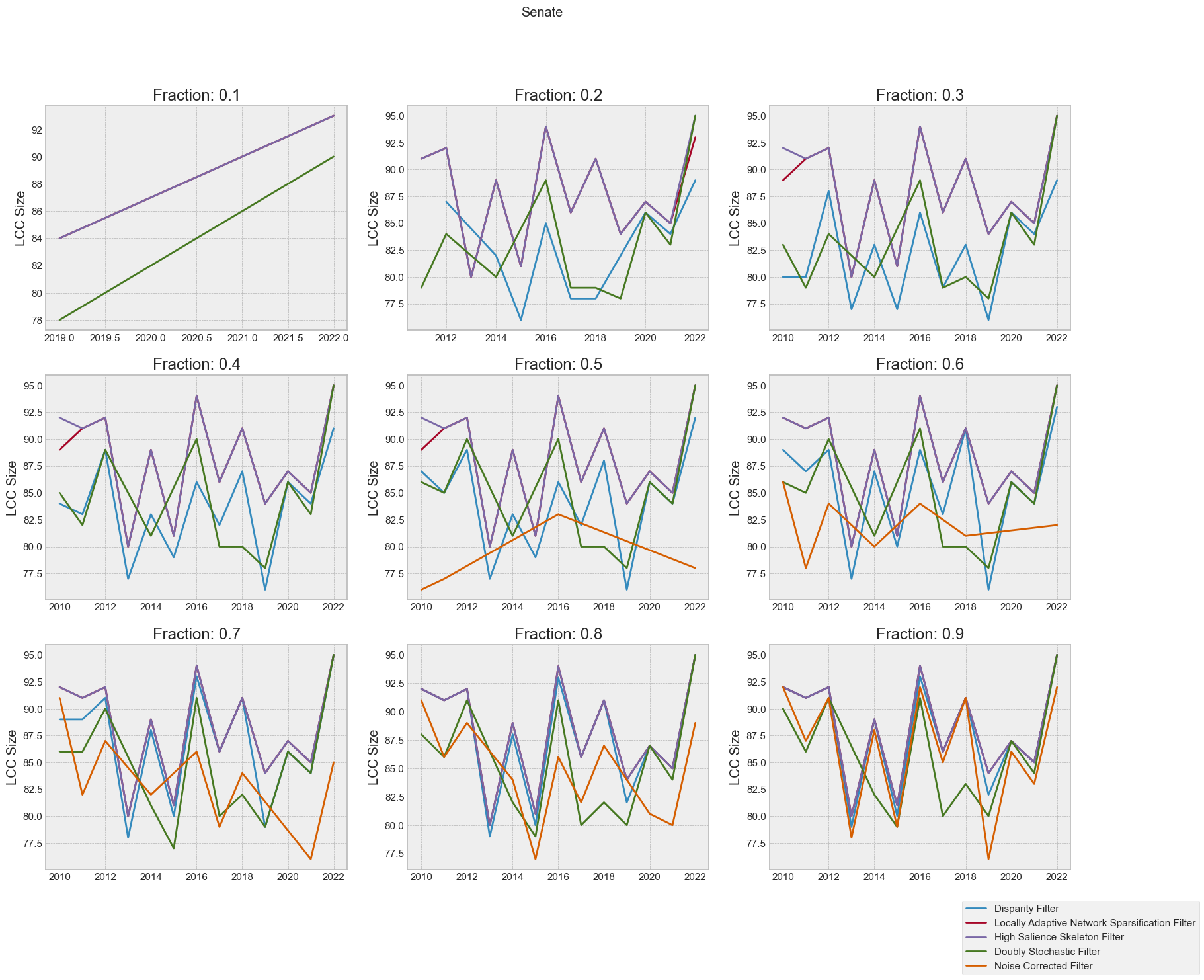

Supplement: S7 Fig — (TIF) [file pone.0319643.s008.tif]

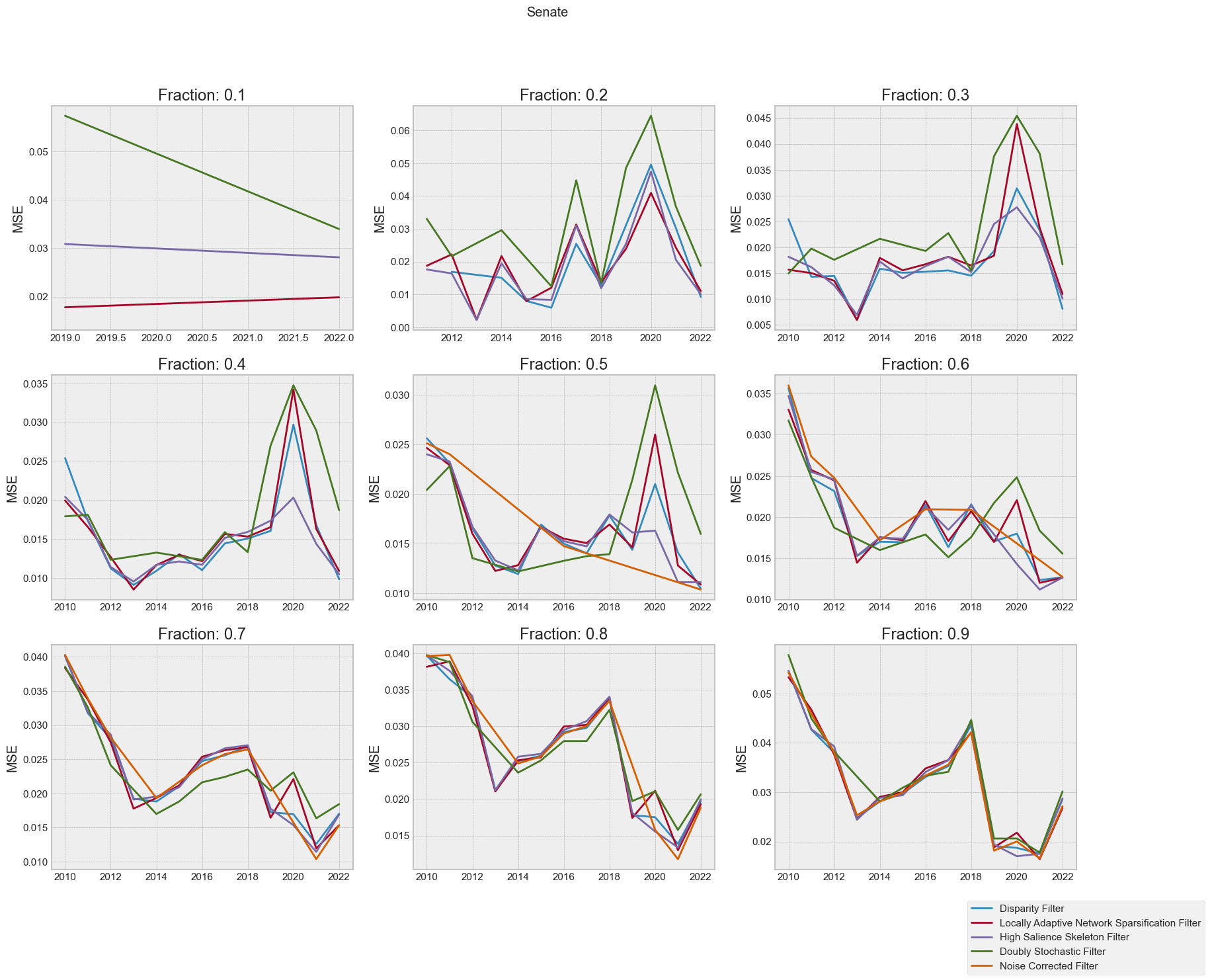

Supplement: S8 Fig — (TIF) [file pone.0319643.s009.tif]

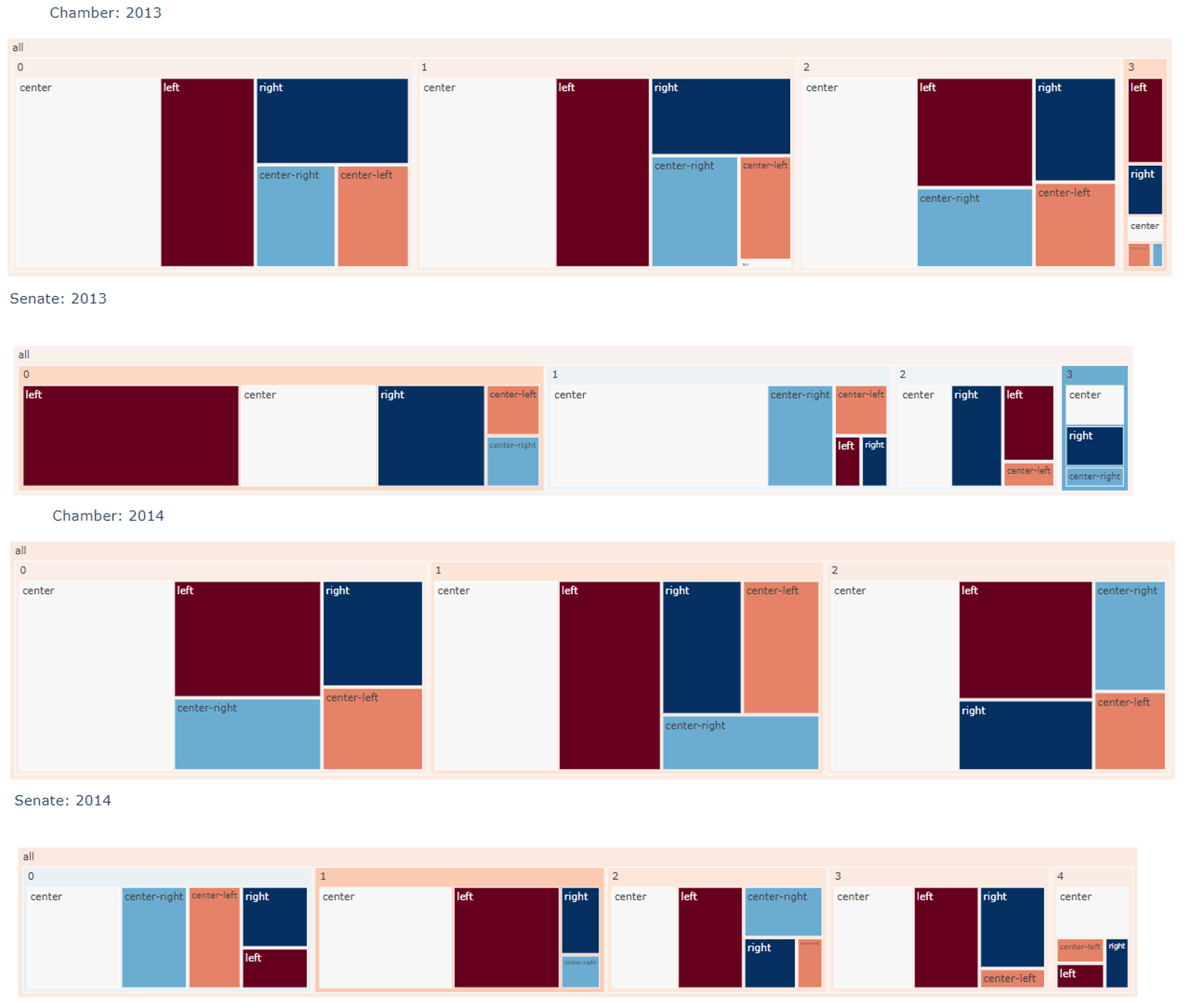

Supplement: S9 Fig — (TIF) [file pone.0319643.s010.tif]

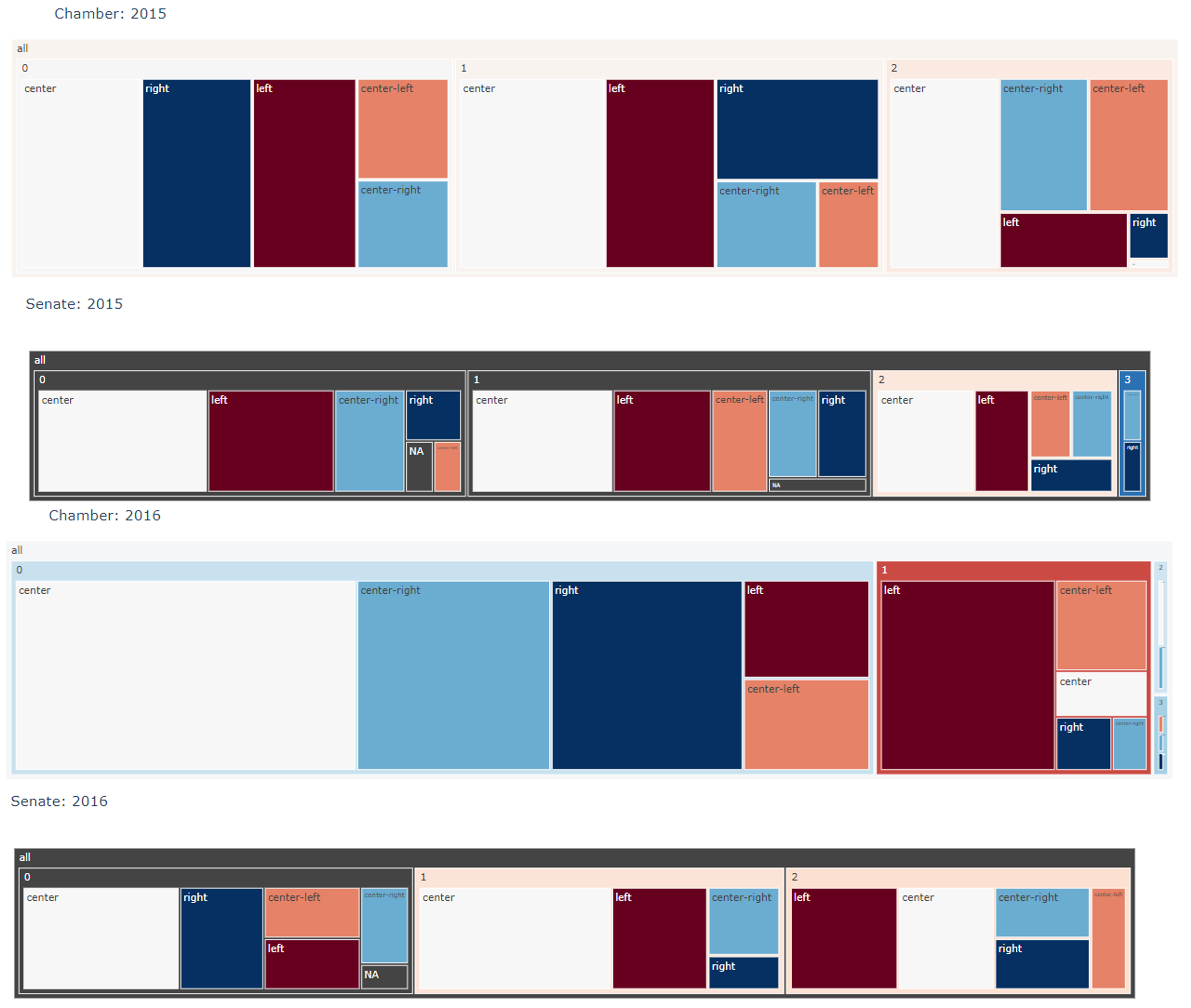

Supplement: S10 Fig — (TIF) [file pone.0319643.s011.tif]
