# Supplementary material for: Complex networks applied to political analysis: Group voting behavior in the Brazilian congress
Source: PLoS One. 2025 Apr 14;20(4):e0319643. doi: 10.1371/journal.pone.0319643 (PMC11996218; doi:10.1371/journal.pone.0319643)
Supplement: Attachment — Submitted filename: PLOS ONE - Rebuttal Letter.pdf [file pone.0319643.s014.pdf]

Rebuttal Letter

Journal Requirements

1. Please ensure that your manuscript meets PLOS ONE's style requirements, including those for file naming.

   A: One instance called Figure was changed for Fig and the calling for several figures was also adjusted. The file names were altered to match the requirement.

2. Please state what role the funders took in the study.
   A: The funders had no role in study design, data collection and analysis, decision to publish, or preparation of the manuscript. This was added to the cover letter.
3. We strongly recommend all authors decide on a data sharing plan before acceptance.

   A: The input data, the processing code to generate results and to generate the rest of the data was made available in GitHub and a link was inserted in the introduction of the paper.

4. Please include captions for your Supporting Information files at the end of your manuscript, and update any in-text citations to match accordingly

   A: Sorry but I did not understand this requirement. Changes were made to the Support Information, please, kindly verify if that is enough.

5. While revising your submission, please upload your figure files to the Preflight Analysis and Conversion Engine (PACE) digital diagnostic tool
   A: As requested, all images were uploaded to PACE and the resulting images were substituted in the LaTeX file.

Reviewer #1

1. In the introduction, the authors state "The political dynamics of a country can be considered a complex system." This general approach has been recognized for years and should be acknowledged (with references) before focusing on the specific topic addressed by the authors.
   A: New references and applications areas were added on the previously paragraph to improve the overall description of the approach. Also, a reference for a survey of applications was introduced to refer the reader for a broader study.
2. The authors state that "the best algorithm for each year and network is different." However, they do not explain the underlying mechanisms behind such finding. A detailed explanation should be provided in the text.
   A: It was clarified that this conclusion was made based on the resulting modularity, Surprise and MSE of the tested backbone extraction methodologies and the appendix containing the overall metrics was referred to.
3. The paper's discussion should expand its scope to elucidate the broader significance of the results. The text should address the following questions, supported by references:
   a. Exactly which findings corroborate previous results in the literature?

b. How does the complex network analysis presented by the authors complement other methodologies used in the analysis of political data in Brazil?
c. What is the overall picture obtained when integrating the authors' findings with the preexisting body of research on political data in Brazil?

A: The final discussion was rewritten to be more explicit about the relationship of the findings when compare to previous body of research.

Reviewer #2

1. The second item listed in the introduction as specific objectives of the work mentions a community detection methodology already proved to work in the chamber of deputies without specifically mentioning which one they are referring to. I should also mention here that, even though a given methodology does return interesting results about something as subjective as identifying political groups, it should hardly be taken as proof.

    A: The item was rewritten to better explain what was done: find the modularity maximization group division. It indeed is not proof that that division is the best assessment of the reality, so this was rewritten as proof that party structure does not maximize modularity.
2. The list below cites the main contributions of the work. The third one (again mentioning a proof), points to modularity. I will in my comments raise many concerns about the use of this quality function. I suggest the authors use modularity and specially the Louvin algorithm very cautiously and making all important caveats, as this function is full of flaws. I point here a few works on its limitations and alternatives (by DOI): 10.1038/s41598-019-41695-z, 10.1073/pnas.0605965104, 10.1103/PhysRevE.81.046106, 10.1371/journal.pone.0024195, 10.1016/j.physa.2022.127063

    A: Following the suggestion, the caveats of the modularity function were made more explicit in the text. Also, the Leiden algorithm was implemented instead of the Louvain algorithm to reduce some of the concerns, although we verified that the results from both algorithms are very similar. Regarding the use of the Surprise metric, it comes with its owns flaws, such as the tendency to generate an exceeding number of groups, which we also verified. This over-division of communities has no backup in the literature which is consistent in finding that, at least of the Chamber of Deputies, the number of effective groups is smaller than the number of parties. Therefore, the selection of the algorithm was used gathering the method combine with the Leiden algorithm with higher Surprise, even with we knowing that the Leiden algorithm will optimize for modularity. We also show that the Surprise of the found communities is also higher than the Surprise of the parties in both houses for every time period.
3. A note on style: I suggest the authors never start a sentence with a citation as done in session Backbone Extraction Methodologies (shouldn't the sessions be numerated?) where the first paragraph reads: "[18] proposed a framework ...".

    A: All sentences with leading citations were rewritten. Also, the overall template and other PLOS ONE publications do not use numerated sections.
4. In the end of this session the authors mention they use Netbone library for the implementation of several methodologies. These several are all the ones used in the present work or others were

used as well? This should be precised and if others were used, please mention where the adopted implementation was taken from.

A: The paragraph was rewritten to make clear that the Netbone has a suite of methods and a subset of those were used in the study and that all methods used were from the library itself.

5. I'm not sure on the origin of the problem, but in my version (downloaded from PLOS ONE server), the figures do not appear in the body of the paper, making its reading very difficult. Next time, before the authors approve the submission, they should make sure all figures are visible in their proper places and not only as stand alone images in the end.

   A: All Figures that appear at the end are in the appendix, therefore they are in the correct position. Also, following the Dr. Hanna Landenmark suggestion: "*Please also note that you may not be able to rearrange the figures to appear in the correct place in the manuscript for the next revision as requested by one reviewer, but that this will also be resolved by the PLOS systems for eventual publication*."

6. In the Backbone Extraction session, the authors start by pointing out what is basically a flaw in the modularity/Louvin algorithm: Namely, that the modularity does not depend solely in the local topological characteristics of the communities, but on the whole network, making the same local topology resulting in different community structures depending on the size of the graph as a whole. Again: be aware of the limitations of Louvin and modularity and how it may impact its use for a specific goal.

   A: Following the answer on question number 2, the caveats were better explicated, the Surprise method was used for the overall selection and the Louvain algorithm was changed to the Leiden algorithm. Both modularity and Surprise of the communities are higher than their respective counterparts in the parties. Again, the maximization of Surprise exacerbates its behavior of over dividing communities, therefore, the Leiden was chosen instead.

7. In Contextual Analysis, I could not really understand what was done. Please, improve the description of the problem at hand you are trying to solve and the solution adopted. Finally here, in the list, I see again that a maximization of modularity is sought. Another important flaw of Q, that may directly hinder its use to divide a parliament into political groups, is the fact that it is biased toward dividing the graph into communities of similar sizes (10.1016/j.physa.2022.127063). There is no reason why all political parties should be of similar sizes.

   A: The entire section on contextual analysis was rewritten to better describe the objective of the analysis and how it was conducted. I believe that the aspects related to modularity were already answered on the points above.

8. In session Government and Opposition the way the communities are chosen as either government and opposition seems fairly arbitrary. Moreover, if the same is done for the chamber of deputies and senate, there is a big problem as the senate is smaller and has, typically, less parties in its composition. For instance in the next session it is mentioned that the senate has typically 9 parties, how are they then divided into government, opposition and neutral if the 5 closest to the government party are the government and the 5 furthest the opposition?

A: Regarding the Senate point, as is shown in Figure 8, the minimum amount of parties is in 2010 and it is equal to 14, not 9. The paragraph says that these 9 parties represent the bulk of over 65% of the senators, not that they are all parties in the house. Regarding the Government and Opposition definitions, the methodology was changed to define each one as the lowest 15% percentile and the highest 15% percentile of the distribution. This definition is still somewhat arbitrary, but allow for a variable number of parties in both groups. We do not have a better cutoff point to propose, neither did we found one adequate in the literature.

9. There are a few instances where a Table is presented as a figure (which I cannot read in my version of the manuscript). Please, make tables as tables, not as figures.

   A: I could not find which cases the reviewer is pointing to. All tables seen defined as tables and there is no image of a table or wrong reference in the paper. Could this be clarified?

10. In session Network Visualization, the last paragraph states that using the same algorithm to both houses can be misleading without some kind of performance test. Well, the thing is that a test can, as well, be biased or subjective, specially if it is modularity based. I would be always inclined to find an algorithm that does work in both cases if I am to compare them both, pointing, whenever the case, to possible caveats. Using many methodologies and selecting the best for each case is a kind of meta analysis of data.

    A: One of the objectives of the work is to find the best backbone extraction algorithm for each house in each year, which we show that, given the metrics we are analyzing, is variable. Both houses present very different topologies and these topologies vary with time since deputies and senators change, as well as political alliances and the executive power. Therefore, we state that using a single algorithm for every time frame and both houses do not take into account these nuances. The idea of doing a meta analysis of the data, which is not actually which is done here, since we are doing algorithm selection, is not detrimental and it is in fact a very common approach in Machine Learning settings that aim to uncover the best method to analyze the data.

11. The next two sessions touch on the subjective matter of political spectrum and parties are labeled as left, right, center, ... Please, state the criteria adopted for these assignments. This is a subjective matter and even though a party may claim to be left or right inclined, once one looks at how their congressmen voted, in most cases they are not really voting coherently in a given subject and may even be divided among communities associated to government and opposition.

    A: The political spectrum, as shown in Table 7 was extracted from the official websites for each party. A paragraph was added to make this clear. As for the idea that a party can define itself as from a side of the spectrum, but not vote in a coherently manner with it, this is true, however, the only way to assert this is to analyze the content of each proposition, which is out of scope for the current work.

12. There are other works that analyse the Brazilian congress, in particular the impeachment period the authors mentioned and analyse, some even pointing to similar conclusions. Please, take a look at 10.1371/journal.pone.0226504 and 10.1016/j.physa.2021.125832, for example.

    A: Both works were added to the discussion and the related work sections. There are probably more works out there with similarities, however, our work does not aim to be a comprehensive systematic review of the entire body of work in the period of the

impeachment. Also, the shown works do not take on the novel approaches of our work, namely: the analysis of the Senate, the comparison between both houses, the systematization and selection of the backbone extraction algorithm, the spectrum analysis of isolation and fragmentation and the government and opposition analysis.
