# Supplementary material for: Complex networks applied to political analysis: Group voting behavior in the Brazilian congress
Source: PLoS One. 2025 Apr 14;20(4):e0319643. doi: 10.1371/journal.pone.0319643 (PMC11996218; doi:10.1371/journal.pone.0319643)
Supplement: Attachment — Submitted filename: Rebuttal Letter - V2 - Revised.pdf [file pone.0319643.s015.pdf]

Rebuttal Letter

Reviewer #3

1. I do not understand the reason why a backbone extraction is performed as a sort of preprocessing step for the community detection, and it makes little sense to me. There is absolutely no problem in running community detection algorithms on the type of provided networks, and there are many other potential algorithms that could be tried, without resorting to first performing a backbone extraction.

    A: The usage of backbone extraction algorithms was used before in several studies involving, not only legislative systems, but overall networks derived from the co-interaction of agents (Ferreira, C. Modeling and analyzing collective behavior captured by many-to-many networks). In this article, we address to expand these previous works by improving the selection of the backbone extraction algorithm, and by expanding it to the Senate, what to the best of our knowledge has not been done before. Networks from agent interactions are prone to the creation of noisy edges, especially in the Legislative System where not all sessions are representative of ideology votes. This is why the backbone extraction methodology was used. Also, several sessions of the paper had additions or were rewritten to better express this rationale.

2. Mucha et. al (2010) proposed a temporal variation of community detection, and also applied it to voting networks in parliament. That approach seems quite well the application of the authors, and is a more reasonable way of getting rid of the “noisy edges” (i.e. some substantial difference need to arise in order to reclassify nodes from one cluster to another, thus reducing the “noise”). Why not use that type of approach? Alternatively, approaches such as Peixoto & Rosvall (2015) seem quite applicable to the type of temporal variation that is of interest to the researchers.

    A: This work aims to improve over previous methodologies commonly applied in the Brazilian Chamber of Deputies by making the whole proposed process more agnostic and data-driven and by expanding the results for the Senate, which was not previously done. The cited studies indeed present methodologies that could be explored to the analysis, and we are now citing them in the next steps as so. However, the objective of this article is not to explore new methodologies and compare with the existing body of work, although we agree that this would be an important study to be investigated. Having other methodologies that can be applied to our problem does not invalidate the chosen methodology, because, in practical terms, for any study there are several methodologies that could qualify. Further, as these methodologies are quite different, direct comparison with previous results from the body of research could be infeasible. It is important to note that the way in which our analysis was performed, by comparing always communities with the parties and how they behave overtime, is not directly translated to a methodology that keeps tracking of nodes (congress members) and parties, which can change overtime.

3. Moreover, the evaluation of what “works” best is rather shallow. That is, the evaluation of the methodology by the provided metrics does not really clarify much. Nor is the evaluation of the

backbone extraction by the maximum modularity very informative. An “expert-based” evaluation of the quality of a partition might be more informative, but this is of course also rather challenging. But why should a backbone extraction approach be better if it results in a higher modularity? This remains unclear to me.

> **A:** An expert-based classification couldn’t be done since we are talking about over 600 congress members each mandate in a period of over 10 years, no expert would have knowledge about so many individuals in order to classify them into groups. The evaluation of the backbone extraction methodology is based on two pillars: reducing the amount of noisy edges, making it easier to visualize and find communities, and to keep the overall phenomenon of the network, which in our case is the alliances between groups, intact.
>
> The first step of the evaluation, called topological analysis, evaluate not only the modularity, but also the Surprise, both which are metrics of how well the found communities (after the application of that backbone extraction algorithm) are, and also the connectivity of the resulting network using the node fraction and the LCC size. This step aims to find the algorithm that is better at uncovering the network structure.
>
> Then, the contextual analysis questioned and developed below and the changes in the paper aims to verify if the resulting network we found has enough remaining data for the phenomenon to be uncovered. This process is measured by the capacity of the remaining data and metadata to predict edge weights.

4. Finally, the authors train a machine learning (ML) model for predicting edge weights (see p. 8, l. 279), but it is not clear to me where this suddenly comes from, nor what the role of that ML model is in the remainder of the paper. Why is this used? What is this used for? How does it relate to the community detection approach?

> **A:** The methodology section of the paper was rewritten to better explain the usage of the ML models. The idea of the contextual analysis is to try and guarantee that the backbone extraction methodology did not pruned edges that are required to explain the phenomenon of interest. To do so, the model is trained to predict edge weights from metadata. If there are enough information remaining in the network to predict these edge weights, it is assumed that the underlying structure was kept intact. Therefore, this is an extra validation step to assess the backbone extraction quality. It does not use any information from any community detection algorithm and therefore is not directly related.

5. Overall then, I’m afraid that I don’t see what the contribution of this paper is. The technical contribution is rather limited and not well motivated. The social scientific contribution about the Brazilian parliament may be more relevant, but given the large role of the technical contribution in the paper, the social scientific contribution seems more marginal. For that reason, I would recommend to reject the paper. It seems that this approach (backbone -> community detection)

has been used before in the same context, and I have my questions about that approach more generally. But even if that approach might make sense, it is not clear to me how the current manuscript shows that the other approaches that the authors consider (i.e., other combinations of backbone / community detection) are "better", and for what reason.

A: Several parts were rewritten to make clear what are the improvements done in the text over previous body of work. They are:

- We show that previous research could be improved by selecting the backbone extraction algorithm using the data, which was not done before.
- We expand the analysis for the Senate, which was done for the first time in Brazil and show how the houses behave differently overtime, especially during important moments of the Brazilian politics. More than that, we also show that the methodology that previous bodies of work used for the Chamber of Deputies can also be applied to the Senate and that many findings stay true, such as the non-maximization of the modularity or Surprise by parties.
- We explore coalitions by using behavioral data, not parties statements with is more accurate to represent what were the true alliances in the Congress.

Therefore, we consider that the paper propose significant improvements over the majority of the body research on the Brazilian Congress. As to why the selected backbone extraction are considered the best, we believe the other answers and changes to the paper shed light on the rationale.

6. p. 5, why only keep edges with weight > 0? The negative edges also provide quite some information about relevant differences, and could inform community detection approaches.

   A: Most of the backbone extraction methodologies used in our work are defined only to a positive edge weight domain. Therefore, to apply and compare the algorithms, only positive edges were kept and edges with weight zero were removed, stating no connection.

7. p. 6, the section on "metric selection for backbone extraction" directly starts with metrics to evaluate the quality of communities, which is rather strange. Please first introduce the backbone extraction itself, and why you end up using community detection methods (which is not clear to me at all)

   A: Following the suggestion, a better description of what is a backbone extraction was added. Also, several parts of the paper were rewritten to better explain the rationale behind the usage of the method.

8. p. 9, the "government" and "opposition" is defined in terms of percentiles. However, it is not clear what percentiles are being calculated, that is on what measure is the distribution (and percentiles) defined? Moreover, this approach does not really seem to make sense to me, but

perhaps there are no clear governing coalitions defined in Brazilian politics, such that another approach is necessary. Without further argumentation this is not clear.

A: In this new version, several sections were rewritten to clarify that we are looking at the percentiles of the coalition distribution between parties. Brazilian parties usually state coalitions and alliances publicly. However, our approach aims to uncover the true alliances, i.e., the real behavior of parties, not what they say they are going to do. To do so we go for a division that is extracted directly from the behavioral data. Furthermore, there are no official channels of coalition statements. Therefore, it is hard to achieve a 'ground-truth' from what parties say their alliances are.
